# 4-(4-Chloro­phen­yl)-5-[1-(4-chloro­phenyl)-2-methyl-2-nitro­prop­yl]-1,2,3-selenadiazole

**DOI:** 10.1107/S1600536807067487

**Published:** 2008-01-04

**Authors:** A. Marx, S. Saravanan, S. Muthusubramanian, V. Manivannan, Nigam P. Rath

**Affiliations:** a Department of Physics, Presidency College, Chennai 600 005, India; b Department of Organic Chemistry, School of Chemistry, Madurai Kamarajar University, Madurai 625 021, India; c Department of Chemistry and Biochemistry, University of Missouri – St Louis, 8001 Natural Bridge Road, St Louis, MO 63121, USA

## Abstract

In the title compound, C_18_H_15_Cl_2_N_3_O_2_Se, the selenadiazole ring makes dihedral angles of 49.87 (3) and 55.70 (3)° with the two benzene rings. The dihedral angle between the two benzene rings is 11.90 (5)°. In the crystal structure, intra­molecular C—H⋯O and C—H⋯Se inter­actions and inter­molecular C—H⋯O, C—H⋯Cl and C—H⋯N inter­actions are observed.

## Related literature

For related literature, see: Bertini *et al.* (1984 ▶); El-Bahaie *et al.* (1990 ▶); El-Kashef *et al.* (1986 ▶); Kuroda *et al.* (2001 ▶); Mellini & Merlino (1976*a* ▶,*b* ▶); Padmavathi *et al.* (2002 ▶); Saravanan *et al.* (2006 ▶); Gunasekaran *et al.* (2007 ▶).
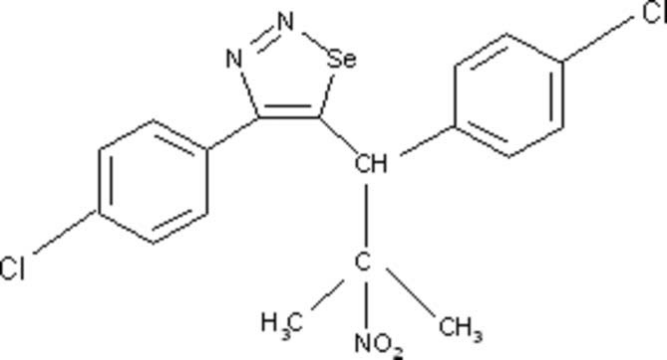


## Experimental

### 

#### Crystal data

C_18_H_15_Cl_2_N_3_O_2_Se
*M* *_r_* = 455.19
Triclinic, 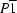

*a* = 7.8352 (2) Å
*b* = 10.9208 (3) Å
*c* = 11.5507 (3) Å
α = 75.381 (1)°
β = 89.044 (1)°
γ = 83.331 (1)°
*V* = 949.80 (4) Å^3^
*Z* = 2
Mo *K*α radiation
μ = 2.27 mm^−1^
*T* = 100 (2) K
0.33 × 0.18 × 0.17 mm

#### Data collection

Bruker Kappa APEXII diffractometer
Absorption correction: multi-scan (**SADABS**; Bruker, 2004 ▶) *T*_min_ = 0.507, *T*_max_ = 0.679
44785 measured reflections
9335 independent reflections
8074 reflections with *I* > 2σ(*I*)
*R*_int_ = 0.028

#### Refinement

*R*[*F* ^2^ > 2σ(*F* ^2^)] = 0.026
*wR*(*F* ^2^) = 0.064
*S* = 1.03
9335 reflections
237 parameters
H-atom parameters constrained
Δρ_max_ = 0.57 e Å^−3^
Δρ_min_ = −0.59 e Å^−3^

### 

Data collection: *APEX2* (Bruker, 2004 ▶); cell refinement: *APEX2*; data reduction: *APEX2*; program(s) used to solve structure: *SHELXS97* (Sheldrick, 1997 ▶); program(s) used to refine structure: *SHELXL97* (Sheldrick, 1997 ▶); molecular graphics: *PLATON* (Spek, 2003 ▶); software used to prepare material for publication: *SHELXL97*.

## Supplementary Material

Crystal structure: contains datablocks global, I. DOI: 10.1107/S1600536807067487/is2267sup1.cif

Structure factors: contains datablocks I. DOI: 10.1107/S1600536807067487/is2267Isup2.hkl

Additional supplementary materials:  crystallographic information; 3D view; checkCIF report

## Figures and Tables

**Table 1:** Hydrogen-bond geometry (Å, °)

*D*—H⋯*A*	*D*—H	H⋯*A*	*D*⋯*A*	*D*—H⋯*A*
C9—H9⋯O2	1.00	2.42	2.8271 (12)	104
C15—H15⋯Se1	0.95	2.86	3.5496 (10)	130
C18—H18*A*⋯Se1	0.98	2.70	3.4209 (10)	130
C7—H7⋯O1^i^	0.95	2.44	3.3757 (13)	167
C15—H15⋯Cl1^ii^	0.95	2.76	3.5923 (10)	147
C17—H17*A*⋯N1^iii^	0.98	2.57	3.4511 (13)	149
C17—H17*A*⋯N2^iii^	0.98	2.60	3.3919 (13)	138

Symmetry codes: (i) 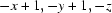; (ii) 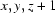; (iii) 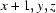.

**Fig. 1.**
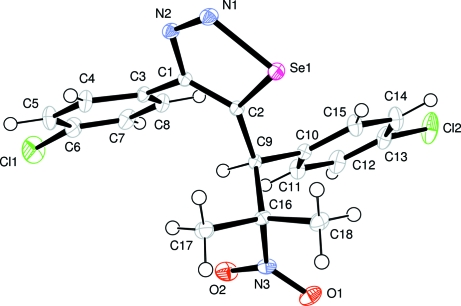
The molecular structure of (I), with atom labels and 50% probability displacement ellipsoids for non-H atoms.

**Fig. 2.**
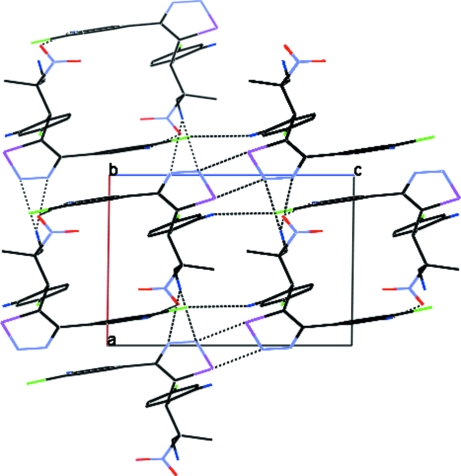
The packing of (I), viewed down the b axis. Hydrogen bonds are shown as dashed lines. H atoms not involved in hydrogen bonding have been omitted.

## supplementary crystallographic information

### Comment

Selenium containing compounds like 1,2,3-selenadiazole possess various beneficial activities like antifungal (Kuroda *et al.*, 2001), antibacterial (El-Kashef *et al.*, 1986), antimicrobial (El-Bahaie *et al.*, 1990) and insecticidal (Padmavathi *et al.*, 2002) activities. As naturally occurring nitro compounds exhibit broad antibiotic activity and certain alkyl nitro compounds exhibit antitumor activity, it was decided to synthesize and structurally characterize a set of 1,2,3-selenadiazoles with nitro group in the side chain (Saravanan *et al.*, 2006).

The geometric parameters in the compound, (I) agree with the reported values of similar structure (Mellini & Merlino, 1976*a*,b; Bertini *et al.*, 1984; Gunasekaran *et al.*, 2007). The C3—C8 benzene ring makes a dihedral angle of 49.87 (3)° with the heterocyclic ring and the C10—C15 benzene ring makes a dihedral angle of 55.70 (3)° with the heterocyclic ring (Fig. 1).

The details of the hydrogen bonding are given in Table 1. The molecular structure is stabilized by weak intramolecular C—H···O and C—H···Se interactions and the crystal packing is stabilized by weak intermolecular C—H···O, C—H···Cl and C—H···N interactions (Fig. 2).

### Experimental

A solution of 2-[1,3-bis(4-chlorophenyl)-4-methyl-4-nitropentylidene]-1-hydrazine carboxamide (0.005 mol) and powdered selenium dioxide (0.05 mol) in dry THF was gently heated on a water bath for 2 h. The selenium deposited on cooling was removed by filtration, and the filtrate was poured into crushed ice, extracted with chloroform, and purified by column chromatography using silica gel (60–120 mesh) with 97:3 petroleum ether: ethyl acetate as eluent to give 4-(4-chlorophenyl)-5-[1-(4-chlorophenyl)-2-methyl-2- nitropropyl]-1,2,3-selenadiazole. Solvent used for crystallization is ethanol.

### Refinement

H atoms were positioned geometrically and refined using riding model, with C—H = 0.95 Å and *U*_iso_(H) = 1.2*U*_eq_(C) for aromatic C—H, C—H = 0.98 Å and *U*_iso_(H) = 1.5*U*_eq_(C) for CH_3_, C—H = 1.00 Å and *U*_iso_(H) = 1.2*U*_eq_(C) for CH.

### Crystal data

**Table:**

C_18_H_15_Cl_2_N_3_O_2_Se	*Z* = 2
*M**_r_* = 455.19	*F*_000_ = 456
Triclinic, *P*1	*D*_x_ = 1.592 Mg m^−^^3^
Hall symbol: -P 1	Mo *K*α radiation λ = 0.71073 Å
*a* = 7.8352 (2) Å	Cell parameters from 8302 reflections
*b* = 10.9208 (3) Å	θ = 2.7–34.5º
*c* = 11.5507 (3) Å	µ = 2.28 mm^−^^1^
α = 75.381 (1)º	*T* = 100 (2) K
β = 89.044 (1)º	Rectangular, colourless
γ = 83.331 (1)º	0.33 × 0.18 × 0.17 mm
*V* = 949.80 (4) Å^3^	

### Data collection

**Table:**

Bruker Kappa-APEXII diffractometer	9335 independent reflections
Radiation source: fine-focus sealed tube	8074 reflections with *I* > 2σ(*I*)
Monochromator: graphite	*R*_int_ = 0.028
*T* = 100(2) K	θ_max_ = 36.7º
ω and φ scans	θ_min_ = 1.8º
Absorption correction: multi-scan(*SADABS*; Bruker, 2004)	*h* = −13→13
*T*_min_ = 0.507, *T*_max_ = 0.679	*k* = −18→18
44785 measured reflections	*l* = −19→19

### Refinement

**Table:**

Refinement on *F*^2^	Secondary atom site location: difference Fourier map
Least-squares matrix: full	Hydrogen site location: inferred from neighbouring sites
*R*[*F*^2^ > 2σ(*F*^2^)] = 0.026	H-atom parameters constrained
*wR*(*F*^2^) = 0.064	*w* = 1/[σ^2^(*F*_o_^2^) + (0.031*P*)^2^ + 0.2243*P*] where *P* = (*F*_o_^2^ + 2*F*_c_^2^)/3
*S* = 1.03	(Δ/σ)_max_ < 0.001
9335 reflections	Δρ_max_ = 0.57 e Å^−^^3^
237 parameters	Δρ_min_ = −0.59 e Å^−^^3^
Primary atom site location: structure-invariant direct methods	Extinction correction: none

### Fractional atomic coordinates and isotropic or equivalent isotropic displacement parameters (Å^2^)

**Table:**

	*x*	*y*	*z*	*U*_iso_*/*U*_eq_	
Se1	0.135681 (12)	0.147694 (9)	0.433060 (8)	0.01400 (3)	
Cl1	0.22480 (5)	0.30162 (4)	−0.32615 (2)	0.03885 (8)	
Cl2	0.23404 (5)	0.83872 (3)	0.28700 (3)	0.03592 (8)	
O1	0.76068 (10)	0.34633 (8)	0.28931 (8)	0.02212 (15)	
O2	0.67115 (11)	0.31526 (8)	0.12446 (7)	0.02317 (16)	
N1	−0.02318 (11)	0.07149 (8)	0.36434 (7)	0.01542 (14)	
N2	−0.01345 (10)	0.10161 (8)	0.25070 (7)	0.01371 (13)	
N3	0.66526 (11)	0.30096 (8)	0.23302 (8)	0.01522 (14)	
C1	0.10681 (11)	0.18154 (8)	0.20053 (8)	0.01114 (14)	
C2	0.20509 (11)	0.21946 (8)	0.27992 (8)	0.01124 (14)	
C3	0.12393 (12)	0.21352 (9)	0.06915 (8)	0.01239 (14)	
C4	0.14006 (15)	0.11629 (10)	0.00999 (9)	0.01870 (18)	
H4	0.1326	0.0309	0.0539	0.022*	
C5	0.16690 (16)	0.14320 (11)	−0.11262 (9)	0.0233 (2)	
H5	0.1771	0.0771	−0.1531	0.028*	
C6	0.17854 (15)	0.26821 (12)	−0.17466 (9)	0.0219 (2)	
C7	0.15700 (15)	0.36712 (10)	−0.11909 (9)	0.02036 (19)	
H7	0.1612	0.4526	−0.1637	0.024*	
C8	0.12902 (13)	0.33887 (9)	0.00348 (8)	0.01577 (16)	
H8	0.1132	0.4058	0.0429	0.019*	
C9	0.35407 (11)	0.29627 (8)	0.24434 (8)	0.01096 (13)	
H9	0.3664	0.3057	0.1563	0.013*	
C10	0.32202 (12)	0.43177 (9)	0.25869 (8)	0.01282 (14)	
C11	0.35859 (14)	0.53150 (9)	0.16337 (9)	0.01794 (17)	
H11	0.4016	0.5133	0.0914	0.022*	
C12	0.33342 (17)	0.65705 (10)	0.17148 (10)	0.0229 (2)	
H12	0.3586	0.7244	0.1059	0.027*	
C13	0.27108 (16)	0.68213 (10)	0.27660 (10)	0.0217 (2)	
C14	0.23240 (16)	0.58569 (10)	0.37302 (10)	0.0226 (2)	
H14	0.1895	0.6045	0.4448	0.027*	
C15	0.25739 (15)	0.46091 (10)	0.36297 (9)	0.01862 (18)	
H15	0.2299	0.3941	0.4284	0.022*	
C16	0.52730 (12)	0.22146 (9)	0.30124 (8)	0.01257 (14)	
C17	0.55570 (13)	0.09225 (9)	0.27148 (10)	0.01807 (17)	
H17A	0.6722	0.0520	0.2961	0.027*	
H17B	0.5412	0.1041	0.1851	0.027*	
H17C	0.4719	0.0377	0.3142	0.027*	
C18	0.55381 (13)	0.20944 (10)	0.43339 (9)	0.01800 (17)	
H18A	0.4704	0.1569	0.4795	0.027*	
H18B	0.5375	0.2943	0.4487	0.027*	
H18C	0.6706	0.1694	0.4575	0.027*	

### Atomic displacement parameters (Å^2^)

**Table:**

	*U*^11^	*U*^22^	*U*^33^	*U*^12^	*U*^13^	*U*^23^
Se1	0.01575 (4)	0.01765 (5)	0.00888 (4)	−0.00696 (3)	0.00236 (3)	−0.00175 (3)
Cl1	0.0533 (2)	0.0539 (2)	0.01020 (10)	−0.01394 (17)	0.00528 (11)	−0.00641 (12)
Cl2	0.0664 (2)	0.01200 (10)	0.02912 (14)	0.00353 (12)	−0.01822 (14)	−0.00708 (10)
O1	0.0197 (3)	0.0181 (3)	0.0286 (4)	−0.0094 (3)	−0.0035 (3)	−0.0024 (3)
O2	0.0213 (4)	0.0293 (4)	0.0161 (3)	−0.0055 (3)	0.0053 (3)	0.0003 (3)
N1	0.0150 (3)	0.0163 (3)	0.0150 (3)	−0.0058 (3)	0.0021 (3)	−0.0024 (3)
N2	0.0135 (3)	0.0140 (3)	0.0138 (3)	−0.0042 (3)	0.0012 (2)	−0.0027 (3)
N3	0.0130 (3)	0.0131 (3)	0.0177 (3)	−0.0032 (3)	0.0011 (3)	0.0003 (3)
C1	0.0116 (3)	0.0109 (3)	0.0108 (3)	−0.0018 (3)	0.0004 (3)	−0.0022 (3)
C2	0.0123 (3)	0.0116 (3)	0.0095 (3)	−0.0024 (3)	0.0007 (3)	−0.0017 (3)
C3	0.0143 (4)	0.0128 (4)	0.0102 (3)	−0.0031 (3)	−0.0005 (3)	−0.0025 (3)
C4	0.0277 (5)	0.0149 (4)	0.0149 (4)	−0.0051 (4)	−0.0010 (3)	−0.0050 (3)
C5	0.0345 (6)	0.0236 (5)	0.0146 (4)	−0.0046 (4)	0.0005 (4)	−0.0094 (4)
C6	0.0267 (5)	0.0298 (5)	0.0097 (4)	−0.0075 (4)	0.0004 (3)	−0.0042 (3)
C7	0.0276 (5)	0.0191 (4)	0.0130 (4)	−0.0084 (4)	−0.0025 (3)	0.0010 (3)
C8	0.0214 (4)	0.0132 (4)	0.0123 (4)	−0.0033 (3)	−0.0020 (3)	−0.0017 (3)
C9	0.0124 (3)	0.0111 (3)	0.0092 (3)	−0.0030 (3)	0.0004 (3)	−0.0016 (3)
C10	0.0156 (4)	0.0110 (3)	0.0113 (3)	−0.0024 (3)	−0.0012 (3)	−0.0013 (3)
C11	0.0264 (5)	0.0128 (4)	0.0137 (4)	−0.0052 (3)	0.0011 (3)	−0.0006 (3)
C12	0.0365 (6)	0.0120 (4)	0.0186 (4)	−0.0052 (4)	−0.0042 (4)	0.0003 (3)
C13	0.0328 (6)	0.0111 (4)	0.0212 (5)	−0.0001 (4)	−0.0095 (4)	−0.0044 (3)
C14	0.0352 (6)	0.0155 (4)	0.0178 (4)	0.0002 (4)	−0.0016 (4)	−0.0068 (3)
C15	0.0285 (5)	0.0138 (4)	0.0134 (4)	−0.0028 (4)	0.0017 (3)	−0.0031 (3)
C16	0.0122 (3)	0.0118 (3)	0.0133 (3)	−0.0045 (3)	0.0007 (3)	−0.0011 (3)
C17	0.0155 (4)	0.0130 (4)	0.0259 (5)	−0.0022 (3)	−0.0007 (3)	−0.0049 (3)
C18	0.0172 (4)	0.0223 (5)	0.0127 (4)	−0.0040 (3)	−0.0029 (3)	−0.0003 (3)

### Geometric parameters (Å, °)

**Table:**

Se1—C2	1.8455 (9)	C9—C10	1.5220 (13)
Se1—N1	1.8652 (9)	C9—C16	1.5630 (12)
Cl1—C6	1.7360 (10)	C9—H9	1.0000
Cl2—C13	1.7350 (10)	C10—C11	1.3918 (13)
O1—N3	1.2216 (12)	C10—C15	1.3944 (13)
O2—N3	1.2247 (11)	C11—C12	1.3889 (15)
N1—N2	1.2734 (11)	C11—H11	0.9500
N2—C1	1.3795 (12)	C12—C13	1.3802 (16)
N3—C16	1.5451 (12)	C12—H12	0.9500
C1—C2	1.3765 (12)	C13—C14	1.3828 (16)
C1—C3	1.4762 (12)	C14—C15	1.3877 (14)
C2—C9	1.5075 (13)	C14—H14	0.9500
C3—C8	1.3923 (13)	C15—H15	0.9500
C3—C4	1.3938 (13)	C16—C18	1.5144 (13)
C4—C5	1.3895 (15)	C16—C17	1.5259 (13)
C4—H4	0.9500	C17—H17A	0.9800
C5—C6	1.3851 (17)	C17—H17B	0.9800
C5—H5	0.9500	C17—H17C	0.9800
C6—C7	1.3826 (16)	C18—H18A	0.9800
C7—C8	1.3901 (14)	C18—H18B	0.9800
C7—H7	0.9500	C18—H18C	0.9800
C8—H8	0.9500		
			
C2—Se1—N1	87.45 (4)	C11—C10—C15	118.31 (9)
N2—N1—Se1	111.08 (6)	C11—C10—C9	118.61 (8)
N1—N2—C1	117.26 (8)	C15—C10—C9	123.07 (8)
O1—N3—O2	123.86 (9)	C12—C11—C10	121.26 (10)
O1—N3—C16	118.80 (8)	C12—C11—H11	119.4
O2—N3—C16	117.33 (8)	C10—C11—H11	119.4
C2—C1—N2	115.83 (8)	C13—C12—C11	118.80 (10)
C2—C1—C3	126.24 (8)	C13—C12—H12	120.6
N2—C1—C3	117.87 (8)	C11—C12—H12	120.6
C1—C2—C9	124.13 (8)	C12—C13—C14	121.63 (10)
C1—C2—Se1	108.37 (6)	C12—C13—Cl2	119.39 (9)
C9—C2—Se1	127.26 (6)	C14—C13—Cl2	118.95 (9)
C8—C3—C4	119.31 (8)	C13—C14—C15	118.74 (10)
C8—C3—C1	121.17 (8)	C13—C14—H14	120.6
C4—C3—C1	119.50 (8)	C15—C14—H14	120.6
C5—C4—C3	120.54 (10)	C14—C15—C10	121.25 (10)
C5—C4—H4	119.7	C14—C15—H15	119.4
C3—C4—H4	119.7	C10—C15—H15	119.4
C6—C5—C4	118.75 (10)	C18—C16—C17	111.81 (8)
C6—C5—H5	120.6	C18—C16—N3	107.47 (8)
C4—C5—H5	120.6	C17—C16—N3	106.56 (7)
C7—C6—C5	121.93 (9)	C18—C16—C9	116.40 (8)
C7—C6—Cl1	118.93 (9)	C17—C16—C9	110.03 (7)
C5—C6—Cl1	119.13 (9)	N3—C16—C9	103.77 (7)
C6—C7—C8	118.62 (10)	C16—C17—H17A	109.5
C6—C7—H7	120.7	C16—C17—H17B	109.5
C8—C7—H7	120.7	H17A—C17—H17B	109.5
C7—C8—C3	120.74 (9)	C16—C17—H17C	109.5
C7—C8—H8	119.6	H17A—C17—H17C	109.5
C3—C8—H8	119.6	H17B—C17—H17C	109.5
C2—C9—C10	114.26 (7)	C16—C18—H18A	109.5
C2—C9—C16	111.87 (7)	C16—C18—H18B	109.5
C10—C9—C16	114.10 (7)	H18A—C18—H18B	109.5
C2—C9—H9	105.2	C16—C18—H18C	109.5
C10—C9—H9	105.2	H18A—C18—H18C	109.5
C16—C9—H9	105.2	H18B—C18—H18C	109.5
			
C2—Se1—N1—N2	−0.27 (7)	Se1—C2—C9—C16	56.27 (10)
Se1—N1—N2—C1	−0.06 (10)	C2—C9—C10—C11	−130.65 (9)
N1—N2—C1—C2	0.52 (12)	C16—C9—C10—C11	98.86 (10)
N1—N2—C1—C3	177.82 (8)	C2—C9—C10—C15	49.27 (12)
N2—C1—C2—C9	174.02 (8)	C16—C9—C10—C15	−81.22 (11)
C3—C1—C2—C9	−3.01 (14)	C15—C10—C11—C12	0.63 (16)
N2—C1—C2—Se1	−0.69 (10)	C9—C10—C11—C12	−179.44 (10)
C3—C1—C2—Se1	−177.72 (7)	C10—C11—C12—C13	0.08 (17)
N1—Se1—C2—C1	0.52 (7)	C11—C12—C13—C14	−0.43 (18)
N1—Se1—C2—C9	−173.98 (8)	C11—C12—C13—Cl2	−178.47 (9)
C2—C1—C3—C8	−50.52 (14)	C12—C13—C14—C15	0.03 (18)
N2—C1—C3—C8	132.50 (10)	Cl2—C13—C14—C15	178.09 (9)
C2—C1—C3—C4	127.69 (11)	C13—C14—C15—C10	0.72 (18)
N2—C1—C3—C4	−49.29 (12)	C11—C10—C15—C14	−1.04 (16)
C8—C3—C4—C5	2.21 (16)	C9—C10—C15—C14	179.04 (10)
C1—C3—C4—C5	−176.03 (10)	O1—N3—C16—C18	−4.50 (11)
C3—C4—C5—C6	0.46 (18)	O2—N3—C16—C18	175.98 (8)
C4—C5—C6—C7	−2.80 (19)	O1—N3—C16—C17	−124.49 (9)
C4—C5—C6—Cl1	176.20 (9)	O2—N3—C16—C17	55.98 (10)
C5—C6—C7—C8	2.35 (18)	O1—N3—C16—C9	119.35 (9)
Cl1—C6—C7—C8	−176.64 (9)	O2—N3—C16—C9	−60.17 (10)
C6—C7—C8—C3	0.43 (16)	C2—C9—C16—C18	−74.54 (10)
C4—C3—C8—C7	−2.67 (15)	C10—C9—C16—C18	57.11 (10)
C1—C3—C8—C7	175.54 (9)	C2—C9—C16—C17	53.95 (10)
C1—C2—C9—C10	111.01 (10)	C10—C9—C16—C17	−174.40 (8)
Se1—C2—C9—C10	−75.30 (10)	C2—C9—C16—N3	167.63 (7)
C1—C2—C9—C16	−117.41 (9)	C10—C9—C16—N3	−60.71 (9)

### Hydrogen-bond geometry (Å, °)

**Table:**

*D*—H···*A*	*D*—H	H···*A*	*D*···*A*	*D*—H···*A*
C9—H9···O2	1.00	2.42	2.8271 (12)	104
C15—H15···Se1	0.95	2.86	3.5496 (10)	130
C18—H18A···Se1	0.98	2.70	3.4209 (10)	130
C7—H7···O1^i^	0.95	2.44	3.3757 (13)	167
C15—H15···Cl1^ii^	0.95	2.76	3.5923 (10)	147
C17—H17A···N1^iii^	0.98	2.57	3.4511 (13)	149
C17—H17A···N2^iii^	0.98	2.60	3.3919 (13)	138

Symmetry codes: (i) −*x*+1, −*y*+1, −*z*; (ii) *x*, *y*, *z*+1; (iii) *x*+1, *y*, *z*.

## References

[bb1] Bertini, V., Dapporto, P., Lucchesini, F., Sega, A. & De Munno, A. (1984). *Acta Cryst.* C**40**, 653–655.

[bb2] Bruker (2004). *APEX2* (Version 1.0–27) and *SADABS* (Version 2004/1). Bruker AXS Inc., Madison, Wisconsin, USA.

[bb3] El-Bahaie, S., Assy, M. G. & Hassanien, M. M. (1990). *Pharmazie*, **45**, 791–793. 2089395

[bb4] El-Kashef, H. S., E-Bayoumy, B. & Aly, T. I. (1986). *Egypt. J. Pharm. Sci.* **27**, 27–30.

[bb5] Gunasekaran, B., Manivannan, V., Saravanan, S., Muthusubramanian, S. & Nethaji, M. (2007). *Acta Cryst.* E**63**, o4024.

[bb6] Kuroda, K., Uchikurohane, T., Tajima, S. & Tsubata, K. (2001). US Patent 6 166 054.

[bb7] Mellini, M. & Merlino, S. (1976*a*). *Acta Cryst.* B**32**, 1074–1078.

[bb8] Mellini, M. & Merlino, S. (1976*b*). *Acta Cryst.* B**32**, 1079–1082.

[bb9] Padmavathi, V., Sumathi, R. P. & Padmaja, A. (2002). *J. Ecobiol.* **14**, 9–12.

[bb10] Saravanan, S., Nithya, A. & Muthusubramanian, S. (2006). *J. Heterocycl. Chem.* **43**, 149–151.

[bb11] Sheldrick, G. M. (1997). *SHELXL97* and *SHELXS97* University of Göttingen, Germany.

[bb12] Spek, A. L. (2003). *J. Appl. Cryst.* **36**, 7–13.

